# The Research of Clinical Decision Support System Based on Three-Layer Knowledge Base Model

**DOI:** 10.1155/2017/6535286

**Published:** 2017-07-27

**Authors:** Yicheng Jiang, Bensheng Qiu, Chunsheng Xu, Chuanfu Li

**Affiliations:** ^1^Centers for Biomedical Engineering, University of Science and Technology of China, Hefei, Anhui 230027, China; ^2^Medical Imaging Center, The First Affiliated Hospital of Anhui University of Chinese Medicine, Hefei 230031, China

## Abstract

In many clinical decision support systems, a two-layer knowledge base model (disease-symptom) of rule reasoning is used. This model often does not express knowledge very well since it simply infers disease from the presence of certain symptoms. In this study, we propose a three-layer knowledge base model (disease-symptom-property) to utilize more useful information in inference. The system iteratively calculates the probability of patients who may suffer from diseases based on a multisymptom naive Bayes algorithm, in which the specificity of these disease symptoms is weighted by the estimation of the degree of contribution to diagnose the disease. It significantly reduces the dependencies between attributes to apply the naive Bayes algorithm more properly. Then, the online learning process for parameter optimization of the inference engine was completed. At last, our decision support system utilizing the three-layer model was formally evaluated by two experienced doctors. By comparisons between prediction results and clinical results, our system can provide effective clinical recommendations to doctors. Moreover, we found that the three-layer model can improve the accuracy of predictions compared with the two-layer model. In light of some of the limitations of this study, we also identify and discuss several areas that need continued improvement.

## 1. Introduction

In the past thirty years, artificial intelligence (AI) has made rapid progress and been widely used in many fields [1]. As an important branch of AI, the concept of the expert system (ES) was introduced in the early 1960s and has also received considerable attention from system researchers and practitioners. In specialized fields, an ES can be used like experts to solve difficult and practical problems. Domain knowledge can be stored in a knowledge base in a specific form. Through interaction with computers, users operate on the knowledge base and systems by using the inference engine within.

Clinical decision support systems (CDSSs) comprise a very active branch of ESs that utilizes medical knowledge engineering. CDSSs make use of ES design principles to simulate the processes of diagnosis and treatment that are usually done by medical experts. The aim is to help doctors solve complicated medical problems or make diagnoses. In return, the medical experts can enrich the knowledge base of the CDSS by sharing their clinical experience and medical knowledge.

Since 1965, the historical development of CDSSs includes systems such as DENDRAL, INTERNIST I, MYCIN, and PUFF [2]; the successful development of these systems demonstrates that ESs have drawn attention widely from the academic and engineering fields. In addition to these large medical expert systems, there are some specialist diagnostic systems developed for particular kinds of diseases. For example, in 2000, Wells et al. developed a knowledge-base system to improve breast cancer treatment [3]. In 2006, Lin et al. developed a decision support system for diagnosis of back pain [4]. In 2012, Anooj proposed a clinical decision support system for cardiac risk prediction based on weighted fuzzy rules [5].

Although CDSSs have been examined in previous research, several challenges still remain [6]. These include representation of the knowledge base, reasoning under uncertainty, and systematic clinical evaluation. Many clinical diagnosis tasks involve reasoning under uncertainty. Researchers believe that intelligent behavior depends on not only the reasoning method but also the knowledge used in the reasoning. In this study, we combined Extensible Markup Language (XML) technology with a professional medical knowledge base and constructed a three-layer model of a professional medical knowledge base. This model expands the knowledge base and utilizes more useful information. The doctors are able to interact with patients online, acquire patient symptom and property information, and input them into our system; the system then calculates the disease and the corresponding probability that the patient may be afflicted. Then, the decision results of the system are compared with actual clinical results for online learning of parameter optimization of the inference engine.

The remainder of this paper is organized as follows. In Section 2, we provide an introduction to a medical knowledge base with a three-layer model. In Section 3, we describe the inference engine of our three-layer model. In Section 4, the parameters of the specificity weighted for online learning are introduced. In Section 5, the system design and its implementation are shown. In Section 6, the system is evaluated and we discuss the results. Conclusions are provided in Section 7.

## 2. Medical Knowledge Base and Its Three-Layer Model

### 2.1. Medical Knowledge Base

For a CDSS to work, it must possess some form of medical knowledge and this knowledge must match the inference engine design principles [7]. Our system's medical knowledge base is built using XML, which is used widely for Web transport; the use of XML provides a unified way to describe and exchange structured data that are independent of the application.

In this study, we built our medical knowledge base by using knowledge from experienced experts and medical literature. Data, information, and knowledge are organized and represented in such a manner that both human and computer are able to understand their meanings [8]. XML uses different labels to describe different kinds of data, which as a type are customized by developers so that they can be extended, modified, or perfected in the future [9]. In this system, two different kinds of knowledge bases are merged into a three-layer model medical knowledge base: a disease knowledge base and a symptom knowledge base for common diseases.

### 2.2. Three-Layer Model

**Figure 1:**
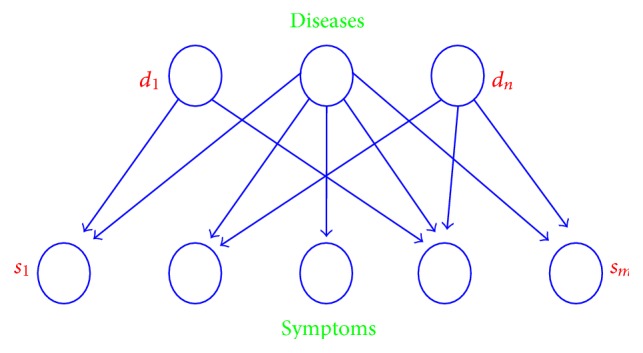
Schematic of “disease-symptom” model.

For a disease, it has many syndromes, such as symptoms and vital signs. Moreover, the same symptoms can occur in different diseases. Take primary bronchial lung cancer and tuberculosis (TB) as an example: patients with either disease would show the same symptoms such as coughing and hemoptysis. We refer to the disease-symptom model as a two-layer model, as shown in Figure 1. In many existing systems, the CDSS is based on a two-layer model of rule reasoning. In other words, the disease is inferred based on the presence of certain symptoms. Using this simple approach makes it difficult to express knowledge accurately when converting that knowledge into machine language such as “IF… AND (OR)… THEN….”

**Figure 2:**
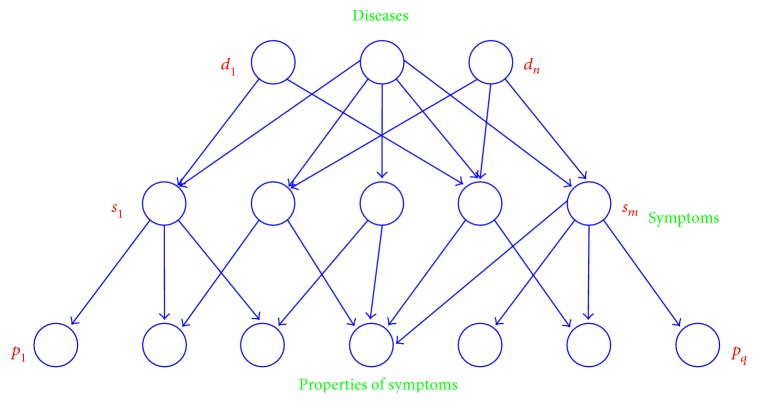
Schematic of “disease-symptom-property” model.

Inspired by the two-layer model and Collins' theory of the decision tree for disease diagnosis [10], we propose a three-layer model of “disease-symptom-property” by adding a “property” to the two-layer knowledge base. For example, TB has a symptom of coughing, for which there are many different properties such as duration and severity of this symptom. Based on this observation, we expand the medical knowledge database to include more details and express the knowledge more accurately. The three-layer model is depicted in Figure 2.

For the inference engine, we need prior probability knowledge. In addition to the presence of symptoms, the properties of symptoms are also included as a prior probability in the disease knowledge base. We assign these prior probabilities to our three-layer model knowledge base by using clinical data and specialist clinical experience. For a disease, the prior probability of this disease is given an initial probability value, and experts also assign the initial probability value of occurrence of different symptoms and properties of the disease. A certain symptom and property combination for a certain specific disease has its specificity, and the occurrence of such a symptom and property leads to the probability of the disease being higher; the “specificity” is then used for weighting of the symptom to diagnose disease in the inference engine.

**Figure 3:**
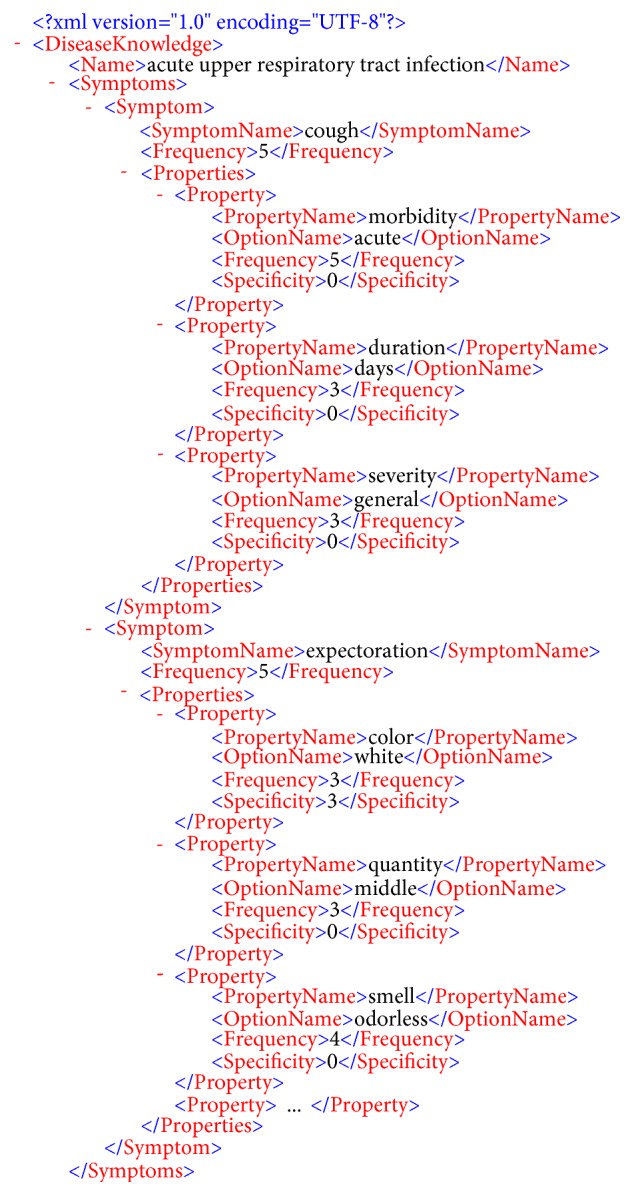
An XML structure model of acute upper respiratory tract infection.

**Figure 4:**
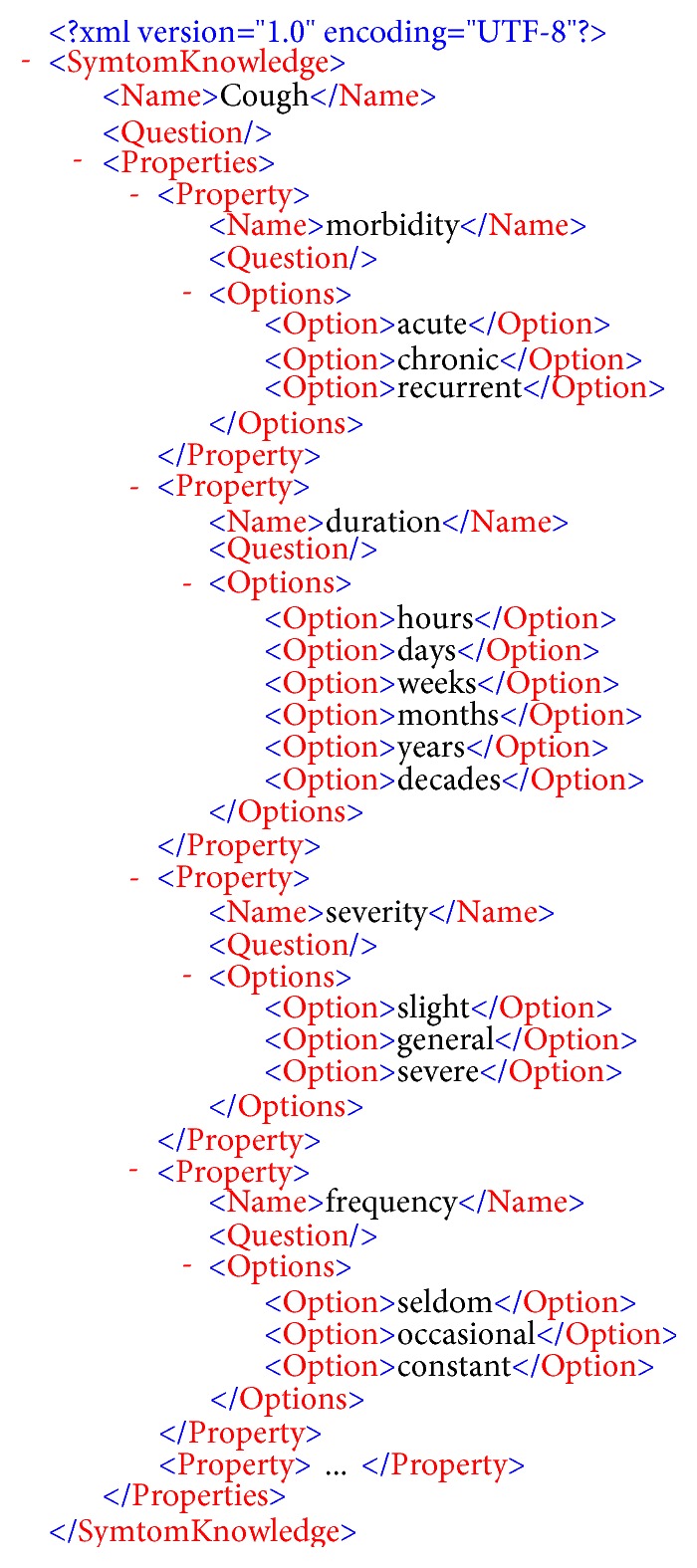
Different properties and options under the cough symptom.

We constructed a three-layer model of an XML medical knowledge base for common respiratory diseases compiled by doctors at the Anhui University of Chinese Medicine. This medical knowledge base includes mainly 11 kinds of respiratory diseases and 380 three-layer model pieces of information, including prior probability knowledge and specificity value.  Figure 3 shows an XML structure model of acute upper respiratory tract infection.  Figure 4 shows the different properties and options under the cough symptom.

From Figure 3, we can see that acute upper respiratory tract infection disease has multiple symptoms. The “frequency” property represents the prior probability knowledge, and the “specificity” property represents a specificity value with a scale of 0–5 to represent the probability value of 0 and 1, respectively.

## 3. Inference Engine: Multisymptom Naive Bayes Algorithm and Symptom Specificity Weighting

### 3.1. Inference Engine

The inference engine preforms the data processing; it is responsible for control and coordination of the entire expert system by using knowledge and applying an inference strategy. The inference engine depends mainly on the representation of internal knowledge. The inference engine of a CDSS can be classified into three types: model-based reasoning, rule-based reasoning, and case-based reasoning (CBR) [2, 11]. Model-based systems simulate the structure and function of the system under study. Rule-based reasoning mainly refers to reasoning based on a series of rules. CBR refers primarily to the use of existing case experience to reason; an example of such a system is Excelicare CBR, a UK commercial clinical decision system [12], which uses electronic medical records as case data for real-time assistance in helping doctors to make decisions.

Decisions are often made by using the probability based on the Bayesian theorem method [13] and belief networks. Developing the inference engine is an important step in constructing a CDSS, and its function is to make decisions and predictions by applying medical knowledge to patients' data. Based on the prior knowledge of Bayes' theorem, the system uses the probability to denote the relation between disease and symptom.

### 3.2. Naive Bayesian Algorithm

The general Bayesian classifier is a kind of classification algorithm that is based on Bayes' theorem. The naive Bayes (NB) algorithm is a very simple, straightforward classification algorithm [14]. In machine learning, naive Bayes classifiers are a family of simple probabilistic classifiers based on applying Bayes' theorem with strong (naive) independence assumptions between the features.

The NB classifier works as follows:

1. As usual, for an *n*-dimensional attribute vector, *X* = (*a*_1_, *a*_2_,…, *a*_*m*_), depicting it from *m* attributes, respectively, *a*_1_, *a*_2_,…, *a*_*m*_.
2. Suppose that there are *n* classes: *C* = (*y*_1_, *y*_2_,…, *y*_*n*_). Given a tuple, *X*, the naïve Bayes algorithm predicts that tuple *X* belongs to class *y*_*i*_, if and only if *P*(*y*_*i*_ | *X*) > *P*(*j* | *X*) (1 ≤ *j* ≤ *n*, *j* ≠ *i*). This is the maximum probable hypothesis and may formally be called the maximum a posteriori hypothesis.
3. Thus, using Bayes' theorem, the conditional probability can be decomposed as

(1) $$\begin{array}{c} P\left(y_{i}\;|\;X\right)=\frac{P\left(X\;|\;y_{i}\right)P\left(y_{i}\right)}{P\left(X\right)}=\frac{P\left(X\;|\;y_{i}\right)P\left(y_{i}\right)}{\sum_{i=1}^{n}P\left(X\;|\;y_{i}\right)P\left(y_{i}\right)}. \end{array}$$

(4) This presumes that the values of the attributes are conditionally independent of one another, given the class label of the tuple. This means that

(2) $$\begin{array}{c} P\left(X\;|\;y_{i}\right)=P\left(a_{1}\;|\;y_{i}\right)P\left(a_{2}\;|\;y_{i}\right)\ldots P\left(a_{m}\;|\;y_{i}\right)=\overset{m}{\underset{j=1}{\prod }}P\left(a_{j}\;|\;y_{i}\right). \end{array}$$

(5) Therefore, formula (1) becomes

(3) $$\begin{array}{c} P\left(y_{i}\;|\;X\right)==\frac{P\left(X\;|\;y_{i}\right)P\left(y_{i}\right)}{\sum_{i=1}^{n}P\left(X\;|\;y_{i}\right)P\left(y_{i}\right)}=\frac{P\left(y_{i}\right)\prod_{j=1}^{m}P\left(a_{j}\;|\;y_{i}\right)}{\sum_{i=1}^{n}P\left(y_{i}\right)\prod_{j=1}^{m}P\left(a_{j}\;|\;y_{i}\right)}. \end{array}$$

### 3.3. Multisymptom Naive Bayes Algorithm

The multisymptom naive Bayes formula is as follows:

(4) $$\begin{array}{c} P\left(D_{i}\;|\;S_{1},S_{2},\ldots ,S_{m}\right)=\frac{P\left(D_{i}\right)\prod_{k=1}^{m}P\left(S_{k}\;|\;D_{i}\right)}{\sum_{i=1}^{n}P\left(D_{i}\right)\prod_{k=1}^{m}P\left(S_{k}\;|\;D_{i}\right)}, \end{array}$$

where

1. *D*_1_, *D*_2_,…, *D*_*n*_ represent kinds of mutually exclusive diseases, with “*i*” representing the sequence number of the disease;
2. *P*(*D*_*i*_) is the prior probability of *D*_*i*_ (the prior probability of occurrence of disease);
3. *S*_1_, *S*_2_,…, *S*_*m*_ are the symptom properties, where “*m*” represents the number of the property;
4. *P*(*S*_*k*_ | *D*_*i*_) is the probability of the occurrence of symptom *S*_*k*_ under disease *D*_*i*_;
5. *P*(*D*_*i*_ | *S*_1_, *S*_2_,…, *S*_*m*_) is the posteriori probability of disease *D*_*i*_ under the condition of the symptoms presented by the patient.

The system first screens out some susceptible factors from the patient information (e.g., gender and age), then combines the remaining information with susceptibility factors in the medical knowledge base (e.g., male common diseases or elderly susceptible to disease), and reports back to the doctor to be asked for related symptoms. Through the interaction between the clinician and the patient, the patient's symptoms are inputted into the system. Under the known condition of prior probabilities, the multisymptom naive Bayes algorithm calculates the posterior probability of the patient's possible disease. The specificity weighting of the symptom is then performed.

### 3.4. Symptom Specificity Weighting

The specificity of these disease symptoms is weighted by an estimation of the degree of contribution to diagnosing the disease. The weighting significantly reduces the dependencies between specificities so that the NB algorithm can be better applied. In 2001, a new model was proposed to improve the NB algorithm by giving a partial weight rather than a standard variable weight value [15]. As stated above, the inference engine of a multisymptom naive Bayes algorithm infers the possible diseases and their corresponding probability, and the current input symptom information determines whether it is specific for the inferred disease. Thus, the corresponding a posteriori probability is weighted as follows:

(5) $$\begin{array}{c} P{\left(D_{i}\;|\;S_{1},S_{2},\ldots ,S_{m}\right)}^{\prime }=P\left(D_{i}\;|\;S_{1},S_{2},\ldots ,S_{m}\right)+P\left(D_{i}\;|\;S_{m}\right)\times \left(1+specificity\;weight\right), \end{array}$$

where *P*(*D*_*i*_ | *S*_1_, *S*_2_,…, *S*_*m*_) are the possible diseases and their corresponding probabilities found by using the multisymptom naive Bayes algorithm, *P*(*D*_*i*_ | *S*_*m*_) is the specificity of the current input symptom to the disease, and *s*pecifity weight ∈ [0, 1) is the specificity weighting value for online learning, whose initial value is 0.6. *P*(*D*_*i*_ | *S*_1_, *S*_2_,…,*S*_*m*_)′ are the calculated disease and the corresponding probability (normalized); this is output along with an explanation of the disease to the Web front-end page.

## 4. Online Learning Process

In the big data era, more and more fields demand high-speed data processing. A large amount of data is required as input for neural network learning and training, especially for the traditional batch machine learning techniques. However, in practice, the limited training data often comes in real time, so the online learning must process the data stream in real time and achieve a balance between speed and accuracy. Routine maintenance and regular updates of the medical knowledge base are necessary. Hence, our online learning system has an advantage in this aspect.

### 4.1. Perceptron Learning Algorithm

In machine learning, the perceptron is an algorithm for supervised learning of binary classifiers (functions that can decide whether or not an input, represented by a vector of numbers, belongs to some specific class). The concept of “reward-punishment” has been widely used in many machine learning algorithms. If the input was classified correctly, the weighting vector does not change. If the input was classified incorrectly, the weight vector will be modified to the proper direction.

The algorithm is as follows:

1. Choose *n* points belonging to the positive or negative instance, in the case of a binary classification problem, as shown in the following type:

(6) $$\begin{array}{c} \begin{array}{c} \left\{\left(x_{1},y_{1}\right),\left(x_{2},y_{2}\right),\ldots ,\left(x_{n},y_{n}\right)\right\},\\ x_{i}=R^{d+1},y_{i}\in \left\{-1,+1\right\}, \end{array} \end{array}$$

where *x*_*i*_ are the corresponding eigenvectors of samples, *y*_*i*_ are the classifications of corresponding sample labels, *d* is the characteristic number, and *n* is the total sample training set. Take the weight vector of the initial value, *w*_0_, beginning at iteration *t* = 1.

(2) Train samples iteratively online, and calculate the values of the weights and the correct vector:

(7) wt=wt−1,wt−1Txi≥0,wt−1+σxi,wt−1Txi<0.

Here, *σ* (*σ* > 0) is an adjustment of the step length.

(3) As long as an error classification remains, return to step 2 until the correct classification for all samples has been achieved.

### 4.2. Online Learning System

This system combines the perceptron theory and the “reward-punishment” concept for specificity weighting parameters for online learning. We compared the clinical results (*D*) of disease diagnosis and the system's results (the highest probability inference results, *D*′); if the clinical results are not consistent with the system's results, we know that the specific weighting parameter has a certain contribution to the algorithm in the inference engine, the online learning method is used to adjust it, and the result will progressively move toward the actual value:

(8) $$\begin{array}{c} Specificity\;weight=\left\{\begin{array}{l} specificity\;weight,D=D^{'},\\ specificity\;weight+\delta \times specificity\;weight,D\neq D^{'}, \end{array}\right. \end{array}$$

where *δ*( = 0.1) is the parameter used for studying; if specificity weight > 1, we assign specificity weight = 0.99.

With the above adjustment parameters, the inference results are converted to the desired result.

## 5. System Design and Its Implementation

Our system was developed using the C# language. The Web, a SQL Server database, and an Internet Information Services server were also used. Users start by registering. There are three kinds of users in the system: common users, managers, and doctors. The system is mainly operated by clicking on a Web page; through the interaction process, suggestions, explanations, and diagnoses can be provided.

### 5.1. System Framework Design

**Figure 5:**
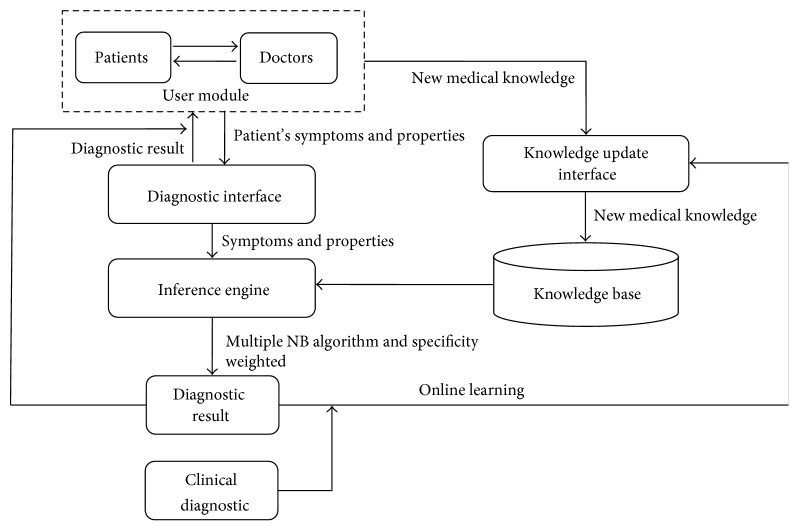
System framework.

As shown in Figure 5, our framework includes four main parts: a user module, an internal inference engine, medical knowledge, and online learning. The user module employs an interactive process in which a doctor selects the patient's symptoms and properties and the system calculates the patient's possible disease information and its probability by using the internal inference engine. The system also includes a knowledge update interface that enables the clinician to update the knowledge base directly. Using this graphical interface, the clinician can add, remove, or modify the three-layer medical knowledge base. The knowledge update interface is an essential part because the clinician may acquire new diagnostic knowledge over time. In the previous section, the other three parts have been described in detail.

### 5.2. Interaction Process

From the system page, the doctor can obtain some basic information about the patient such as name, sex, and age. The system sorts out several susceptible factors, such as gender-susceptible factors (common diseases for males or females), age-susceptible factors (age-related disease, and so forth), initial feedback about possible symptoms of diseases, and common disease options. In the future, susceptibility factors will include information from outpatient departments. After the doctor selects the symptoms and properties, the page will asynchronously input the symptoms into our internal reasoning algorithm and calculate the patient's possible disease information and its probability. The page will refresh a new symptomatic problem and show the probability of the disease through a chart to the doctor. The explanatory module will display an explanation of the possible disease. Doctors and patients continue to interact and repeat the process iteratively.

**Figure 6:**
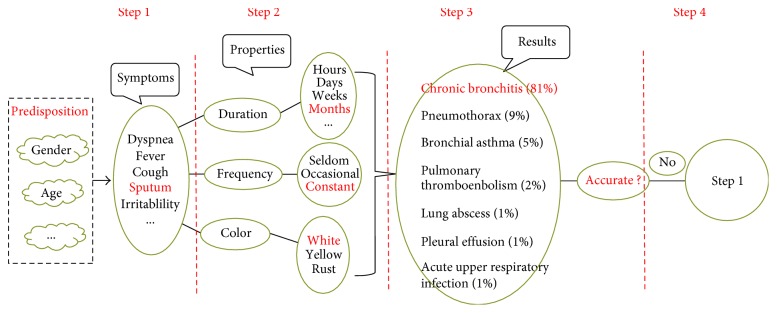
Interaction diagram of the system.

Figure 6 shows an interactive process diagram. In step 1, a list of symptoms, obtained through a number of susceptible factors, is displayed. In step 2, the properties and their options under the corresponding symptomatic list are displayed. In step 3, the possible diseases and their corresponding probabilities calculated by the inference engine algorithm are displayed; if the results are not reliable, the process is repeated from step 1.

## 6. System Evaluation and Results

Our system evaluation includes clinical efficacy and comparison with a two-layer system using real-world testing cases. First, two senior specialist doctors tested the application of the system and gave us some comments on the front-end page that enabled us to modify the system and simplify its operation. Second, doctors validated the clinical efficacy by using 50 clinical cases, including 10 kinds of respiratory disease; these cases were commonly used to validate knowledge-based systems for demonstrating whether a system exhibits a performance level comparable to that achieved by human experts. Finally, we built a knowledge base based on a two-layer model to compare with the three-layer model.

### 6.1. Clinical Efficacy and Results

Doctors simulated an interaction scenario with patients, clicking on the page to select the patient's symptoms and properties, then inputting them into the internal inference engine. The system inference results and clinical diagnosis results were analyzed, and we used five measurements to distinguish the results in our clinical efficacy evaluation: deterministic, recommended, suggested, possible, and recall. The deterministic type means that the probability of the correct disease being derived by the system is between 0.8 and 1; it measures the system's power and particularly emphasizes diagnostic success. For the recommended and suggested types, the probabilities are between 0.6 and 0.8 and between 0.4 and 0.6, respectively; these two types indicate the portion of a gold standard diagnosis (which may consist of multiple parts) that has been correctly recommended by the system. For the possible type, the probability is defined as 0.1 to 0.4 and, for the recall type, the probability of disease is less 0.1. The results are summarized in Table 1.

The system results of recall type offer no decision-making suggestions for doctors. We can define a clinical misdiagnosis proportion as

(9) $$\begin{array}{c} misdiagnosis\;proportion\;\left(\%\right)=\left(\frac{N_{recall}}{N_{test}}\right)\times 100\%. \end{array}$$

By using Table 1, we can calculate misdiagnosis proportion (%) = (*N*_recall_/*N*_test_) × 100% = (1/50) × 100% = 2% , and so correct proportion (%) = 1 − misdiagnosis proportion (%) = 98%. Otherwise, we found that most of the results are distributed in the range of recommended and suggested types; this conforms to the actual situation, because the system diagnoses the disease by means of an interaction with patients and provides some recommendations and suggestions to assist the clinician in making clinical decisions, but other auxiliary examinations are also required to diagnose the disease, such as imaging examination and blood tests. Of course, if a certain symptom has its specificity for a specific disease in our three-layer model knowledge base, the probability of being afflicted with this disease will be relatively large, so the system will give a deterministic result like a deterministic type.

### 6.2. Comparison with Two-Layer Model and Results

A medical knowledge base based on a two-layer model cannot express complete knowledge; thus, inferring disease just from the presence of symptoms is not always accurate. For example, coughing, presence of sputum, fever, and other symptoms are used to diagnose TB, but these symptoms can also occur in acute upper respiratory tract infection or bronchiectasis disease. We added a “property” (e.g., duration and severity) to this two-layer model to better distinguish these similar diseases, and these properties have their own specificity for specific disease. Doctors sorted out the symptom information based on a two-layer model from clinical cases, and they input them into the internal inference engine. The results are given in Table 2, and Table 3 compares the misdiagnosis proportions of two- and three-layer models.

We analyzed the results of Table 2 through a chi-square (*χ*^2^) test [16]. Since *χ*^2^ = 22.123 > *χ*_0.05,4_^2^ = 9.49, *p* = 0.00019 < 0.05, there is a significant statistical difference. Thus, by using Tables 2 and 3, we found that most of the results are distributed into the possible type based on the two-layer model, so this model cannot provide effective clinical recommendations to doctors, and the two-layer model also has a lower correct proportion and higher misdiagnosis proportion than the three-layer model.

## 7. Conclusions

In this paper, we have proposed a system based on a three-layer model that can calculate the posterior probability of the patient's possible disease. The three-layer model knowledge base utilizes more useful information in inference and can effectively solve the expression inaccuracy of the knowledge by adding the “property” to the two-layer knowledge base. For online learning, the decision results of our three-layer model were compared with actual clinical results to train the parameters of the inference engine. By evaluation, we found that our system can provide effective clinical recommendations to doctors.

Our current system is limited to common diseases found in respiratory medicine, and we need to expand it and include vital signs, laboratory and radiographic knowledge, and so forth. The NB classifier is one of most effective classification models, but it is based on the attribute independence assumption; however, this assumption is often violated in real-world data-mining applications. There are many optimization algorithms that can be used to improve the accuracy of the NB classifier by weakening its attribute independence assumption; these optimization algorithms include lazy Bayesian rules [17], tree-augmented naive Bayes (TAN) [18], and super-parent TAN [19], which led to the development of the NB classifier. There have been some new efficient techniques to improve computational efficiency too, such as hidden naive Bayes [20], averaged one-dependence estimators [21], weighted average of one-dependence estimators [22], randomly selected naive Bayes [23], discriminatively weighted naive Bayes [24], and deep feature-weighted naive Bayes [25]. In the future, we will focus on how to further improve the accuracy and efficiency of the inference engine algorithm.

## Figures and Tables

**Table 1:** Summary of clinical efficacy evaluation results.

	Deterministic	Recommended	Suggested	Possible	Recall	Sum
Probability distribution	0.8–1	0.6–0.8	0.4–0.6	0.1–0.4	<0.1	
Test case	7	13	24	5	1	50
Overall performance	0.14	0.26	0.48	0.10	0.12	1.00

**Table 2:** Comparison with two-layer model.

	Deterministic	Recommended	Suggested	Possible	Recall	Sum
Probability distribution	0.8–1	0.6–0.8	0.4–0.6	0.1–0.4	<0.1	
Three layers	7	13	24	5	1	50
Two layers	2	7	13	22	6	50
Sum	9	20	37	27	7	100

**Table 3:** Correct proportion and misdiagnosis proportion.

	Correct proportion	Misdiagnosis proportion
Three-layer model	98%	2%
Two-layer model	88%	12%
